# Population Biology and Epidemiological Studies of *Acinetobacter baumannii* in the Era of Whole Genome Sequencing: Is the Oxford Scheme Still Appropriate?

**DOI:** 10.3389/fmicb.2020.00775

**Published:** 2020-04-29

**Authors:** Xiaoting Hua, Linyue Zhang, Jintao He, Sebastian Leptihn, Yunsong Yu

**Affiliations:** ^1^Department of Infectious Diseases, Sir Run Run Shaw Hospital, Zhejiang University School of Medicine, Hangzhou, China; ^2^Key Laboratory of Microbial Technology and Bioinformatics of Zhejiang Province, Hangzhou, China; ^3^Zhejiang University-University of Edinburgh Institute, Zhejiang University School of Medicine, Hangzhou, China

**Keywords:** *Acinetobacter baumannii*, whole genome sequencing, cgMLST, mlst, genotyping

Recently, discussions arose about the accuracy of the two *Acinetobacter baumannii* multilocus sequence typing (MLST) Schemes (Hamidian et al., 2017; Castillo-Ramirez and Grana-Miraglia, 2019; Gaiarsa et al., 2019). Santiago et al. showed that neither the Oxford scheme nor the Pasteur scheme could reflect the relationships among *A. baumannii* isolates accurately (Castillo-Ramirez and Grana-Miraglia, 2019). Stefano Gaiarsa et al. reported issues of the Oxford scheme regarding *gdhB* paralogy, recombination, primer sequences, and position of the genes on the genome (Gaiarsa et al., 2019). The importance of using more powerful genotyping strategies is highlighted when analyzing bacteria with highly dynamic genomes like *Acinetobacter baumannii*. The decrease in costs of whole genome sequencing would make the technology an ideal tool for genotyping of bacterial species. Here, we provide experimental data obtained from the two methods, allowing a direct comparison between the core genome MSLT (cgMLST) and core SNP (cgSNP) with the MLST.

First, we revisited the genomic sequences of 172 previously described *A. baumannii* isolates that were obtained from three hospitals in Hangzhou, China (Hua et al., 2017). Six isolates that did not belong to *A. baumannii* were excluded. The aim was to construct robust phylogenetic trees of the 166 genome sequences by using MLST, cgMLST and cgSNP data individually, and to then determine the differences and similarities between the two methods. To avoid confusion, we used ST_oxf_ and ST_pas_ to distinguish Oxford scheme and Pasteur scheme in *A. baumannii* MLST. The MSLT results showed that our datasets covered 20 ST_oxf_s and 10 ST_pas_s_._ Compared to the Oxford scheme (ST_oxf_), there were fewer STs in the Pasteur scheme (ST_pas_) (Figure 1A). The diversity of the two MLST schemes were assessed using the Simpson's Diversity Index with a 95% confidence interval (http://www.comparingpartitions.info/). For the Pasteur scheme, the Simpson's Diversity Index is 0.291 (0.202–0.380) while it is 0.823 (0.781–0.866) for the Oxford scheme. The Oxford scheme was more discriminatory than the Pasteur scheme. The result of the Oxford scheme, which displayed a higher Simpson's Diversity Index, is confirmed by Gaiarsa et al. who based their calculation on the genomes of the three International Clones using 730 genomes (Gaiarsa et al., 2019). We used the genome dataset used in Bautype which contained all the available genome assemblies from Genbank (3519 as of May, 2019) to confirm the comparative result of the Oxford scheme and the Pasteur scheme (Hua et al., 2020). For the Pasteur scheme, the Simpson's Diversity Index is 0.645 (0.626–0.664) while it is 0.934 (0.929–0.940) for the Oxford scheme.

**Figure 1:**
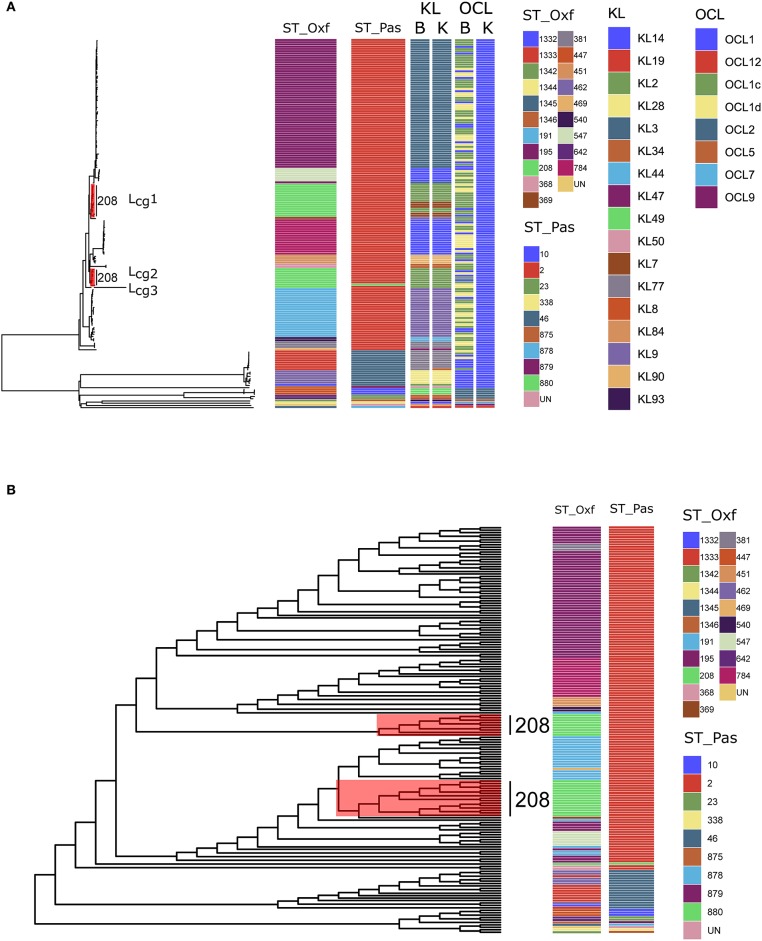
**(A)** Maximum-likelihood phylogeny reflecting the relationships among *Acinetobacter baumannii* isolates via cgMLST. The corresponding MLST types, KL types, and OCL types are shown on the right. Three distinct lineages (L_cg_1, 2, 3) from ST_oxf_208 were marked with red. ST_Oxf: types defined by the Oxford scheme; ST_Pas: types defined by the Pasteur scheme; B: types defined by Bautype; K: types defined by Kaptive; **(B)** Maximum-likelihood phylogeny via cgSNP. The corresponding MLST types, are shown on the right. Three distinct lineages (Lcg1, 2, 3) from ST_oxf_208 were marked with red.

In the first step of our work, among 166 isolates, 145 isolates were assigned to 11 different STs by the Oxford scheme but only one ST (ST_pas_ 2) when using the Pasteur scheme. This observation provides further support for the opinion that was put forward by Santiago et al. who elaborates that the two schemes show different levels of resolution with the Pasteur scheme, revealing considerably less detail than the Oxford scheme when distinguishing between *A. baumannii* isolates (Castillo-Ramirez and Grana-Miraglia, 2019). This observation also confirmed the result of Gaiarsa et al. that the Oxford MLST scheme has higher discriminatory power and higher concordance than Pasteur MLST (Gaiarsa et al., 2019).

Second, we performed a genome-wide gene-by-gene comparison by employing the cgMLST target definer function of the software Ridom SeqSphere+, version 5.1.0 (Ridom, Münster, Germany) using default parameters (Junemann et al., 2013). We constructed a maximum-likelihood phylogeny via 2390 core genes to determine the differences between the isolates. The cgMLST method showed higher Simpson's Diversity Index (0.964, CI: 0.951–0.976) than the Pasteur scheme and the Oxford scheme. Based on our cgMLST profiles, all STs in either scheme formed coherent lineages except for ST_oxf_208. Surprisingly, we found that ST_oxf_208 strains could be divided into three distinct lineages (named as L_cg_1, 2, 3) rather than subdivided internally (Figure 1A). This phenomenon indicated that the internal differences determined by cgMLST of these 24 strains were greater than the differences determined by the seven housekeeping genes applied in the Oxford scheme. The capsular polysaccharide (KL) and lipooligosaccharide outer core (OCL) were detected by Kaptive (Wyres et al., 2020) and Bautype (Hua et al., 2020). For the KL characterization, results from Kaptive and Bautype correlate, except for XH662. The KL type of XH662 in Kaptive is KL34, while it is KL77 in Bautype. The different predicted KL type was caused by the difference among reference sequences of KL77 used in Kaptive and Bautype. The sequence of KL77 in Kaptive has one more A at the position 21925 compared to that in Bautype. KL recombination was observed in L_cg_1 (Figure 1A). There were two KLs, KL2 and KL7 in L_cg_1. For OCL, all ST208 belonged to OCL1 when performing the analysis using Kaptive. The Bautype result showed that OCL1, OCLc, and OCLd appeared in ST208 strains. The difference in OCL results indicated a different OCL database curator strategy in Kaptive vs. Bautype.

Next, we constructed phylogenetic tree base core genome SNPs. Core genome SNPs were called for each isolate using Snippy v4.4.5 (https://github.com/tseemann/snippy), and each isolate was mapped to the reference genome XH386 (CP010779.1). Alignments were filtered for recombinations using Gubbins v2.3.4 (Croucher et al., 2015). A maximum likelihood tree was inferred with RAxML v8.2.12 under the GTRCAT model (Stamatakis, 2014). The phylogenetic trees were visualized using ggtree v1.16 (Yu, 2020). The result of cgSNP also support the finding of cgMLST that ST_oxf_208 strains could be divided into three distinct lineages (Figure 1B).

Last, in order to investigate the reasons that might cause this phenomenon, we analyzed the variable allelic genes in cgMLST within ST_oxf_208 strains and defined them as two gene sets. The first gene set contained 11 adjacent genes and formed two different allelic variation combinations, based on which ST_oxf_ 208 strains could be roughly divided into 2 groups (L_cg_2 as a group1, L_cg_1 and L_cg_3 as group2). The second gene set contained 14 genes distributed discretely on the chromosome and formed three different allelic variation combinations that could further distinguish L_cg_1 and L_cg_3 from the previously described “group 2.” We also found the same allelic variation combinations in the first gene set appearing in other non-ST_oxf_208 strains as well (Table 1). For example, ST_oxf_208 L_cg_2 strains and a ST_oxf_381 strain (XH663) shared the same allelic variation combination. Similarly, ST_oxf_208 L_cg_1, L_cg_3 strains and a ST_oxf_191 strain (XH697) exhibited the same phenomenon. Considering the involved genes were adjacent, we speculate that this is due to homologous recombination, a mechanism which was proposed previously (Castillo-Ramirez and Grana-Miraglia, 2019). A previously published report describes that the *gpi* gene, which is used in the Oxford scheme, is located near the capsule biosynthesis gene cluster, with recombination replacements of this cluster region being common, again possibly due to homologous recombination. As a result, a different *gpi* sequences were introduced, leading to problems for typing (Hamidian et al., 2017). This illustrates that a more suitable MLST scheme, including more reliable housekeeping genes, needs to be developed.

**Table 1:** The cgMLST and MSLT results of all ST_oxf_208 strains and two non-ST_oxf_208 strains.

	**Sample**	**XH506**	**XH507**	**XH546**	**XH549**	**XH550**	**XH684**	**XH686**	**XH687**	**XH727**	**XH728**	**XH748**	**XH804**	**XH823**	**XH835**	**XH839**	**XH667**	**XH671**	**XH672**	**XH698**	**XH706**	**XH733**	**XH747**	**XH780**	**XH794**	**XH663**	**XH697**	**Functions of the corresponding genes**
	SToxf	208	208	208	208	208	208	208	208	208	208	208	208	208	208	208	208	208	208	208	208	208	208	208	208	381	191	
	STpas	2	2	2	2	2	2	2	2	2	2	2	2	2	2	2	2	2	2	2	2	880	2	2	2	2	2	
	STcg	1069	1069	1076	710	1077	710	710	710	1101	1076	825	710	825	710	710	1088	716	716	716	1099	1104	1108	716	UN	1087	1095	
	Lcg								1											2					3	/	/	
	OCL(B)	OCL1	OCL1c	OCL1c	OCL1c	OCL1c	OCL1d	OCL1d	OCL1d	OCL1c	OCL1c	OCL1	OCL1c	OCL1c	OCL1c	OCL1c	OCL1	OCL1d	OCL1	OCL1c	OCL1c	OCL1d	OCL1	OCL1c	OCL1d	OCL1d	OCL1d	
	OCL(K)	OCL1	OCL1	OCL1	OCL1	OCL1	OCL1	OCL1	OCL1	OCL1	OCL1	OCL1	OCL1	OCL1	OCL1	OCL1	OCL1	OCL1	OCL1	OCL1	OCL1	OCL1	OCL1	OCL1	OCL1	OCL1	OCL1	
	KL(B)	KL7	KL7	KL7	KL2	KL2	KL2	KL2	KL2	KL7	KL7	KL2	KL2	KL2	KL2	KL2	KL2	KL2	KL2	KL2	KL2	KL2	KL2	KL2	KL2	KL77	KL9	
	KL(K)	KL7	KL7	KL7	KL2	KL2	KL2	KL2	KL2	KL7	KL7	KL2	KL2	KL2	KL2	KL2	KL2	KL2	KL2	KL2	KL2	KL2	KL2	KL2	KL2	KL77	KL9	
The first gene set	ACICU_RS04540	4	4	4	4	4	4	4	4	4	4	4	4	4	4	4	1	1	1	1	1	1	1	1	4	1	4	Hypothetical protein
	ACICU_RS04550	4	4	4	4	4	4	4	4	4	4	4	4	4	4	4	12	12	12	12	12	12	12	12	4	12	4	Porin
	ACICU_RS04555	4	4	4	4	4	4	4	4	4	4	4	4	4	4	4	11	11	11	11	11	11	11	11	4	11	4	Membrane protein
	ACICU_RS04560	4	4	4	4	4	4	4	4	4	4	4	4	4	4	4	11	11	11	11	11	11	11	11	4	11	4	Dihydrodipicolinate synthase family protein
	ACICU_RS04565	6	6	6	6	6	6	6	6	6	6	6	6	6	6	6	12	12	12	12	12	12	12	12	6	12	6	Transcriptional regulator
	ACICU_RS04610	4	4	4	4	4	4	4	4	4	4	4	4	4	4	4	10	10	10	10	10	10	10	10	4	10	4	Hypothetical protein
	ACICU_RS04615	6	6	6	6	6	6	6	6	6	6	6	6	6	6	6	12	12	12	12	12	12	12	12	6	12	6	Membrane protein
	ACICU_RS04620	6	6	6	6	6	6	6	6	6	6	6	6	6	6	6	13	13	13	13	13	13	13	13	6	13	6	Alpha/beta hydrolase
	ACICU_RS04625	6	6	6	6	6	6	6	6	6	6	6	6	6	6	6	13	13	13	13	13	13	13	13	6	13	6	Arginine:ornithine antiporter
	ACICU_RS04630	4	4	4	4	4	4	4	4	4	4	4	4	4	4	4	11	11	11	11	11	11	11	11	4	11	4	Homocysteine S-methyltransferase
	ACICU_RS04635	11	11	11	11	11	11	11	11	11	11	11	11	11	11	11	12	12	12	12	12	12	12	12	11	12	11	Hypothetical protein
The second gene set	ACICU_RS12120	NA	NA	59	59	59	59	59	59	NA	NA	59	NA	59	59	59	NA	1	1	1	1	1	1	NA	1	1	1	Alpha/beta hydrolase
	ACICU_RS02045	10	10	10	10	10	10	10	10	10	10	10	10	10	10	10	1	1	1	1	1	1	1	1	33	1	33	Magnesium transporter
	ACICU_RS03980	6	6	6	6	6	6	6	6	6	6	6	6	6	6	6	1	1	1	1	1	1	1	1	6	1	6	Cell division protein ZipA
	ACICU_RS08385	11	11	11	11	11	11	11	11	11	11	11	11	11	11	11	1	1	1	1	1	1	1	1	1	1	1	Transcriptional regulator
	ACICU_RS08715	12	12	12	12	12	12	12	12	12	12	12	12	12	12	12	1	1	1	1	1	1	1	1	1	1	1	Hypothetical protein
	ACICU_RS08800	67	67	67	67	67	67	67	67	67	67	67	67	67	67	67	1	1	1	1	1	1	1	1	1	1	1	NAD(FAD)-dependent dehydrogenase
	ACICU_RS12140	26	26	26	26	26	26	26	26	NA	NA	26	NA	26	26	26	NA	1	1	1	1	1	1	NA	1	1	1	Hypothetical protein
	ACICU_RS12165	56	56	56	56	108	56	56	56	NA	NA	56	56	56	56	56	1	1	1	1	1	1	1	NA	1	1	1	Acyl-CoA dehydrogenase
	ACICU_RS12175	64	64	64	64	64	64	64	64	64	64	64	64	64	64	64	1	1	1	1	1	1	1	NA	1	1	1	Amino acid ABC transporter permease
	ACICU_RS12510	6	6	6	6	6	6	6	6	6	6	6	6	6	6	6	1	1	1	1	1	1	1	1	6	1	6	Nickel transporter
	ACICU_RS13390	49	49	49	49	49	49	49	49	49	49	49	49	49	49	49	1	1	1	1	1	1	1	1	1	1	1	Phenazine biosynthesis protein PhzF
	ACICU_RS13865	42	42	42	42	42	42	42	42	42	42	42	42	42	42	42	1	1	1	1	1	1	1	1	1	1	1	MFS transporter
	ACICU_RS14495	6	6	6	6	6	6	6	6	6	6	6	6	6	6	6	1	1	1	1	1	1	1	1	6	1	6	Hydrolase
	ACICU_RS15635	67	67	67	67	67	67	67	67	67	67	67	67	67	67	67	1	1	1	1	1	1	1	1	1	1	1	Acyl-CoA dehydrogenase

*The variable allelic genes applied in cgMLST within ST_oxf_208 strains and their functions are listed. SToxf, types defined by the Oxford scheme; STpas, types defined by the Pasteur scheme; STcg, types defined by cgMLST; Lcg, lineages of ST_ox_208 strains determined by cgMLST; NA, notapplicable*.

*A. baumannii* has emerged as a critical pathogen of nosocomial infections worldwide, particularly in intensive care (Wong et al., 2017). ST_oxf_208 was identified as predominant ST of carbapenem resistant *A. baumannii* in China (Deng et al., 2014). The MLST scheme has been used for infectious disease epidemiology, revealing the relationships within and between bacterial lineages. Although MLST has many advantages over other molecular typing methods, it cannot reflect the true relationships of isolates for *A baumannii* using only a small number of chromosomal segments due to the high levels of recombination events in the bacterial genome (Castillo-Ramirez and Grana-Miraglia, 2019). Our analysis demonstrated that strains of the predominant type ST_oxf_208 could be divided into three distinct lineages when employing cgMLST and cgSNP, which showed a superior discriminatory ability compared with the conventional MLST technique, where all strains are clustered together as the same type. By using the information from cgMLST and/or cgSNP, we might be able to gain a better perspective to understand the epidemiology of *A. baumannii* infections in China, but also globally.

## Author Contributions

XH and YY designed the study. LZ and XH analyzed the bioinformatics data. LZ, JH, and SL wrote the manuscript.

## Conflict of Interest

The authors declare that the research was conducted in the absence of any commercial or financial relationships that could be construed as a potential conflict of interest.
